# Genotype–Phenotype Relationship among 785 Unrelated White Women with Inherited Congenital Factor VII Deficiency: A Three-Center Database Study

**DOI:** 10.3390/jcm13010049

**Published:** 2023-12-21

**Authors:** Susan Halimeh, Lydia Koch, Gili Kenet, Piotr Kuta, Tess Rahmfeld, Monika Stoll, Ulrike Nowak-Göttl

**Affiliations:** 1 Coagulation Center Rhine-Ruhr, 47051 Duisburg, Germany; susan_halimeh@gmx.de; 2 Institute of Clinical Chemistry, University Hospital of Kiel & Lübeck, 23538 Lübeck, Germany; lydia.koch@uksh.de (L.K.); p.kuta@uke.de (P.K.);; 3 Thrombosis Unit, National Hemophilia Center, Tel Hashomer and the Sackler Medical School, Tel Aviv University, Tel Aviv 69978, Israel; gili.kenet@sheba.health.gov.il; 4 Institute of Human Genetics, Genetic Epidemiology, University of Muenster, 48149 Muenster, Germany; mstoll@uni-muenster.de; 5 Department of Biochemistry, Genetic Epidemiology and Statistical Genetics, Maastricht University, 6211 LK Maastricht, The Netherlands

**Keywords:** rare coagulation disorders, factor VII deficiency, genotype–phenotype relationship

## Abstract

Background: Congenital factor VII (FVII) deficiency, a rare bleeding disorder resulting from mutations in the F7 gene with autosomal recessive inheritance, exhibits clinical heterogeneity that lacks a strong correlation with FVII:C levels. The objective of this study was to discern genetic defects and assess their associations with the clinical phenotype in a substantial cohort comprising 785 white women exhibiting FVII:C levels below the age-dependent cut-off percentage. Patients and Methods: Individuals with verified inherited factor VII deficiency underwent i) genotyping using the Sanger method and multiplex ligation-dependent probe amplification (MLPA) to identify F7 mutations, including common polymorphic variants. Additionally, they were ii) categorized based on clinical bleeding scores (BS). Thrombophilic variants and blood groups were also determined in the study participants. Results: The probands in this study encompassed both asymptomatic individuals (referred for a laboratory investigation due to recurrent prolonged prothrombin time; n = 221) and patients who manifested mild, moderate, or severe bleeding episodes (n = 564). The spectrum of bleeding symptoms included epistaxis, gum bleeding, gastrointestinal bleeding, hematuria, postoperative bleeding, and gynecologic hemorrhage. The median ISTH bleeding score (BS) recorded within a two-year period prior to the work-up was 2 (0–17). Notably, this score was significantly higher in symptomatic women compared to their asymptomatic counterparts (3 versus 0; *p* < 0.001). The corresponding PBAC score before hormonal treatment stood at 225 (5–1200), exhibiting a positive correlation with the ISTH BS (rho = 0.38; *p* = 0.001). Blood group O was more prevalent in symptomatic women compared to asymptomatic individuals (58 versus 42%; *p* = 0.01). Among the 329 women (42%), known and novel mutations in the F7 gene, encompassing coding regions, exon/intron boundaries, and the promoter region, were identified, while common polymorphisms were detected in 647 subjects (95%). Logistic regression analysis, adjusted for clinical and laboratory data (including blood group, FVII activity, the presence of F7 gene mutations and/or polymorphisms, thrombophilia status, and additional factor deficiencies) revealed that older age at referral (increase per year) (odds/95% CI: 1.02/1.007–1.03), the presence of blood group O (odds/95% CI: 1.9/1.2–3.3), and the coexistence of further bleeding defects (odds/95% CI: 1.8/1.03–3.1) partially account for the differences in the clinical bleeding phenotype associated with FVII deficiency. Conclusion: The clinical phenotype in individuals with FVII deficiency is impacted by factors such as age, blood group, and the concurrent presence of other bleeding defects.

## 1. Introduction

Factor VII (FVII) deficiency is characterized by a bleeding disorder linked to FVII coagulant activity falling below 50% of the normal level [1]. The estimated prevalence of FVII deficiency typically ranges from 1 in 300,000 to 500,000 individuals [2,3].

The perception of FVII deficiency as a mild bleeding disorder arises from its classification as an umbrella term, encompassing asymptomatic forms of this autosomal recessively inherited disease [2].

Both genders exhibit symptoms of FVII deficiency, including epistaxis, bruising easily, bleeding gums, muscle hematoma, hemarthrosis, gastrointestinal bleeding, hematuria, central nervous system (CNS) bleeding, and postoperative bleeding. In women, there is an additional association with heavy menstrual bleeding (HMB) [3].

Following their investigation into the correlation between genetic factors and clinical presentation in FVII deficiency, Mariani et al. (2005) [3] proposed a severity classification system for this condition:

(i) Severe forms, characterized by central nervous system (CNS) and/or gastrointestinal (GI) bleeding and/or hemarthrosis;
(ii) Moderate forms, defined by three or more symptoms, excluding CNS and/or GI bleeding and/or hemarthrosis;
(iii) Mild forms, exhibiting one or two symptoms, primarily muco-cutaneous ones, and excluding CNS and/or GI and/or hemarthrosis [1].

Their research found that 28.8% of patients fell under the ‘severe’ category, 26.5% were classified as ‘moderate’, and 44.8% were identified as ‘mild’ bleeders [1].

Currently, two main international registries exist for factor VII (FVII) deficiency: the International Registry on Congenital Factor VII Deficiency (IRF7) [4] and the Seven Treatment Evaluation Registry (STER) [5]. In 2013, an analysis of these registries, which included 785 FVII deficiency cases, revealed that about 20% of the patients experienced severe, life-threatening FVII deficiency or suffered major disabilities as a result. Research by Di Minno et al. in 2013 based on data from these registries indicated that bleeding associated with this condition can begin at any age and that clinical presentation generally remains consistent throughout a patient’s life [5].

Bernardi and Mariani’s 2020 review [1] on the biochemical, molecular, and clinical facets of FVII deficiency highlights a persistent ambiguity, despite over 70 years of research. Specifically, the connection between the genotype and clinical phenotype of FVII deficiency is not well understood. This uncertainty has resulted in paradoxical cases where individuals with extremely low FVII levels exhibit no symptoms while others with only moderately decreased FVII levels experience severe bleeding [1].

In their 2000 study, Millar et al. [6] investigated the genotype–phenotype relationship in a group of unrelated patients with FVII deficiency. They discovered that the variability in laboratory phenotype observed among individuals could not be entirely explained by the presence of different F7 gene lesions or their combinations. Notably, the variation in antigen levels among heterozygous family members of the probands was significantly greater between different families than within the same family. Similarly, inter-familial differences in the activity levels of the heterozygous relatives were more pronounced than intra-familial variations. These observations led the authors to conclude that the laboratory phenotype is primarily influenced by the specific F7 gene lesion present in the family. However, they found no correlation between laboratory phenotype and polymorphism genotype. As a result, Millar et al. recommended conducting larger-scale studies to further explore the genotype–phenotype relationship in FVII deficiency [6]. Consequently, our study aims to undertake such a large-scale investigation and identify the underlying factors that influence the relationship between laboratory and bleeding phenotypes in FVII deficiency.

### 1.1. Patients and Methods

#### 1.1.1. Ethics

The underlying multicenter cohort study was approved by the medical ethics committee of the University of Münster & Kiel [B304/16], Germany, and written informed consent was provided in all cases prior to study participation.

**Figure 1:**
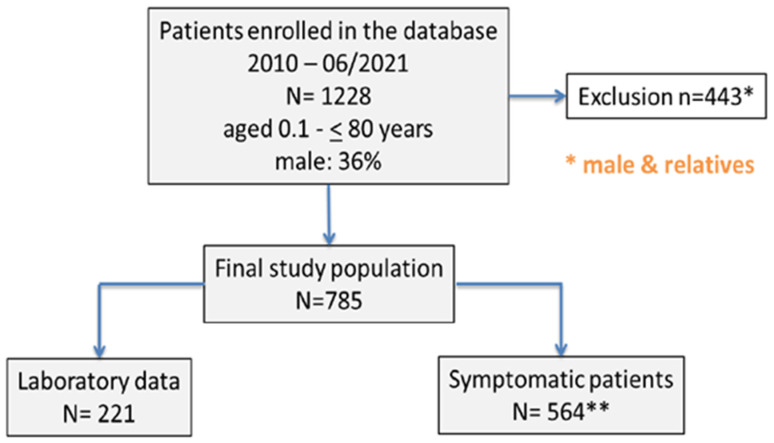
Patient flow chart. ** Number of patients enrolled and excluded in the study were depicted.

This study’s data were obtained from a database initially comprising 1228 male and female patients with FVII deficiency. These patients, ranging in age from 0.1 to 80 years, were associated with three coagulation treatment centers located in Duisburg, Lübeck, and Kiel, Germany. For our analysis, we first excluded 28 relatives, and subsequently 415 males, from the database, meaning that the final study cohort included n = 785 women with FVII deficiency (Figure 1).

#### 1.1.2. Laboratory Analysis

On the patients enrolled in the database, we performed a plasma-based analysis of residual FVII coagulant (FVII:C) using a clotting-based assay in the form of a modified PT-method with recombinant human tissue factor (TF) using the ACL TOP 750. Congenital platelet disorders were ruled out by impedance aggregometry (Multiplate^®^, Multiplate Roche Diagnostics, Mannheim, Germany) and in vitro-bleeding time (PFA-200^®^, PFA Siemens Healthineers, Marburg, Germany).

#### 1.1.3. Genotyping

In this study, genetic analyses were conducted on patients with confirmed Factor VII deficiency. Genomic DNA was extracted from whole blood samples using QIAGEN DNA extraction kits. For F7-genotyping, DNA amplification was carried out via polymerase chain reaction (PCR), followed by bidirectional sequencing using the Sanger method on the 3730xl DNA Analyzer from Applied Biosystems, Darmstadt, Germany. Primers were designed based on the genomic reference sequence NM_000131.4 from the GeneBank database. We examined the promoter region, nine exons of the F7 gene [7], and their adjacent non-coding areas for sequence variations, including common polymorphisms. Major deletions were also assessed using Multiplex Ligation Dependent Probe Amplification (MLPA) with the P207-kit from MRC Holland.

The genetic variants identified were analyzed using Alamut Visual 2.15 (SOPHiA GENETICS), a software that consolidates data on variant characteristics such as location, predicted protein change, and references in databases and literature. Additionally, the variants were checked in the EAHAD Coagulation Factors Database (https://f7-db.eahad.org/, accessed on 23 November 2023) [8].

Furthermore, the patients were tested for common thrombophilic sequence variations using custom TaqMan SNP genotyping assays (ThermoFisher, Waltham, MA, USA) on the 7900 HT Fast Real-Time PCR system (Applied Biosystems, Waltham, MA, USA). These tests included factor V Leiden (rs6025), prothrombin G20210A (rs1799963) and A19911G (rs3136516), MTHFR C677T (rs1801133), and PAI-1 promoter 4G/5G (rs1799768).

#### 1.1.4. Determination of Bleeding Phenotypes

To assess the bleeding phenotypes of our patients, we utilized two distinct instruments: the Bleeding Assessment Tool (BAT) developed by the International Society on Thrombosis and Hemostasis (ISTH) [9], which was applied to both male and female patients, and the Pictorial Blood Loss Assessment Chart (PBAC) [10].

#### 1.1.5. Data Analysis

In our analysis, we employed several statistical methods. The Wilcoxon–Mann–Whitney U test and the Kruskal–Wallis test were used for calculations involving continuous data. For comparing frequency distributions, we applied the chi-square test and Fisher’s exact test. To determine correlations, the Spearman rank test was utilized. Additionally, odds ratios (OR), along with 95% confidence intervals (CI), were estimated using a logistic regression model.

## 2. Results

In this cohort study, continuous data are presented as mean and standard deviation. Out of the 785 female patients enrolled, 564 (71.85%; median [range] age 33.5 years [18–87]) were symptomatic, while 221 (28.15%; median [range] age 31 years [18–81]) were asymptomatic (as illustrated in Figure 1). The median [range] FVII plasma activity was 49.5% [5–57%] in asymptomatic cases versus 51% [5–60%] in women with symptomatic bleedings (*p* = 0.04). The bleeding symptoms observed in these women included menorrhagia (40%), skin bleeding such as recurrent hematomas (25.6%) and/or petechiae (23%), both-sided epistaxis (22%), major surgery-associated hemorrhage (16.4%), and postpartum hemorrhage (14%). In addition, bleeding following tooth extraction (6%), gum bleeding (4%), and bleeding following minor surgery (3.3%) were documented, as well as gastrointestinal bleeding (0.8%) and joint hemorrhage (0.4%).

The median International Society on Thrombosis and Hemostasis (ISTH) bleeding score (BS), recorded over a two-year period prior to evaluation, was 2, ranging from 0 to 17. This score was notably higher in symptomatic patients compared to asymptomatic ones (3 vs. 0; *p* < 0.001). In women, the median Pictorial Blood Loss Assessment Chart (PBAC) score prior to hormonal treatment was 225, ranging from 5 to 1200, showing a positive correlation with the ISTH BS (rho = 0.38; *p* = 0.001). Additionally, blood group O was found to be more frequent in symptomatic female patients compared to asymptomatic ones (58% vs. 42%; *p* = 0.01).

In our study, mutations in the F7 gene, encompassing coding regions, exon/intron boundaries, and the promoter region, were identified in 329 of the women (42%). Among these, a total of 354 mutations were observed, with 94 distinct variants being identified. Notably, 30 of these variants were neither recorded in the EAHAD F7-database [8] nor previously mentioned in the literature (as detailed in Table 1). Fifty-two patients exhibited a combination of two or three mutations.

The majority of these mutations were missense variants, accounting for 67.8%. Other types of mutations included small deletions (3.11%), frameshift variants (6.78%), nonsense variants (1.13%), splice site variants (11.02%), intronic single-nucleotide variations (3.39%), and promoter single-nucleotide variations (5.93%). Additionally, one synonymous variant (0.28%) and complete deletion of the F7 gene (0.56%) were detected. It is important to note that mutations previously reported by our team are listed in a Supplementary Table S1. Common F7 gene polymorphisms, found in 647 subjects (95%), are not separately depicted.

In our study, we found no significant difference in the mutation profile of the F7 gene when comparing the symptomatic and asymptomatic patients (*p* = 0.27). However, the presence of at least one F7 mutation was more frequently associated with lower factor VII (FVII) activities (*p* < 0.001).

Through a logistic regression analysis, adjusted for clinical and laboratory data (including blood group, FVII activity, the presence of F7 gene mutations and/or polymorphisms, thrombophilia status, and other factor deficiencies), we identified several factors that could explain the differences in the clinical bleeding phenotypes associated with FVII deficiency. These factors included older age at referral (with an increased odds per year, odds/95% CI: 1.02/1.007–1.03), the presence of blood group 0 (odds/95% CI: 1.9/1.2–3.3), and the coexistence of additional bleeding defects (odds/95% CI: 1.8/1.03–3.1). Of note, the presence of thrombophilia did not explain the bleeding phenotype in the present study (odds/95% CI: 0.77/0.44–1.35). These variables collectively accounted for 73.18% of the variance in clinical bleeding phenotype.

Almost half of the 785 F VII deficiency patients (48.66%) had at least one additional coagulation disorder. Table 2a,b show the numbers and percentages of patients with disorders other than VWD as secondary and/or further bleeding disorders. Interestingly, two of the patients with additional bleeding disorders also suffered from Noonan Syndrome, which is a non-coagulation disorder. None of the patients suffered from congenital platelet disorders, had a combination of additional bleeding disorders, or had additional thrombophilia.

Apart from hemorrhagic basis diseases, 189 of the 785 females (24.59%) had at least one additional thrombophilic state. As depicted in Supplementary Table S2, the most common additional thrombophilia was FV Leiden, followed by Prothrombin G20210A mutation, elevated lipoprotein (a), homozygous MTHFR C677T TT mutation, and Prothrombin A19911G polymorphism. None of the patients with thrombophilia had an additional bleeding disorder other than FVII deficiency.

The percentage of thrombophilia (n = 189/24.59%) was equally distributed among women studied.

## 3. Discussion

In our female cohort with factor VII deficiency, our multivariable analysis revealed that the three factors determining the bleeding phenotype in FVII deficiency were age, blood group 0, and concomitant bleeding defects. Taken together, however, the listed factors are by no means associated with FVII deficiency as a bleeding disorder only. In addition, the results obtained from genotyping did not explain the differences between symptomatic and asymptomatic FVII-deficient patients.

Age at first bleeding episode is a significant predictor of disease severity in both Hemophilia and von Willebrand disease (VWD). In the realm of Hemophilia research, several studies have corroborated this correlation. Hang et al. (2011) [11] conducted a study on 56 boys with Hemophilia A and confirmed that an earlier first joint bleed was a crucial indicator of a more severe bleeding phenotype in this condition. Similarly, Den Uijl et al. (2011) analyzed data from 377 boys with Hemophilia A and found a direct correlation between baseline factor VIII (FVIII) levels and age at first bleeding. They observed that joint bleeding commenced at a significantly younger age in patients classified as having severe hemophilia according to the ISTH severity classification compared to those with moderate hemophilia. The study also noted that patients with mild hemophilia experienced the onset of joint bleeding at an older age [12].

In another relevant study, Goren et al. (2020), with a cohort of 55 boys with Hemophilia A, demonstrated that the age at first joint bleed was a strong predictor of subsequent joint damage [13]. These findings from various studies highlight the importance of early bleeding episodes as a prognostic factor in Hemophilia A.

The diagnosis of von Willebrand disease (VWD) in patients under two years old is challenging, leading to a scarcity of research on infants and toddlers with this condition [14]. As a result, data linking age at first bleeding with the prognosis of VWD are limited. However, there have been some notable studies in this area.

Dupervile et al. (2021) conducted a study with 105 patients under the age of two diagnosed with VWD, finding that 70% of them experienced their first bleeding episode [14]. Additionally, Siboni et al. (2016) [15] examined a cohort of 103 patients with extremely low levels of VWF:RCo (<6 IU dL^−1^). They discovered that baseline factor VIII (FVIII) levels were correlated with age at first bleeding symptoms in patients with extremely low levels of VWF, except for those with types 2A and 2B VWD [15].

These findings suggest that both factor VII (F VII) and factor VIII (F VIII) demonstrate a similar correlation between age at first bleeding and the severity of the later phenotype. This parallel suggests that early bleeding episodes may be a critical indicator of disease severity in both hemophilia and VWD.

In alignment with the findings from our cohort, Dentali et al. (2013) [16] conducted a meta-analysis encompassing 22 studies with 9468 bleeding patients and over 450,000 controls. This comprehensive analysis revealed a slight but statistically significant increase in bleeding risk among patients belonging to the O blood group compared to those from non–O blood groups [16]. The clinical significance of this association is heightened by the fact that approximately 45% of the general population possesses the O blood group, resulting in a population-attributable risk of 12.7% [16].

The issue of bleeding is particularly relevant in women due to factors such as menstrual bleeding and obstetric complications. Generally, women experience bleeding more frequently than men, leading to a higher likelihood of being referred for bleeding problems and requiring treatment due to sex-specific bleeding phenotypes [17]. In a study by Atiq et al. (2021) [17], the most commonly reported symptoms in women over the age of 12 were heavy menstrual bleeding and postpartum hemorrhage (PPH) [17]. These observations highlight the gender-specific aspects of bleeding disorders and the importance of considering these differences in clinical evaluations and treatments.

The most extensive literature review on the co-occurrence of factor VII (FVII) deficiency with other inherited bleeding disorders was conducted by Girolami et al. in 2007 [18]. Their review encompassed 14 papers published between 1955 and 1999, detailing a total of 25 cases where FVII deficiency was associated with various clotting defects, specifically deficiencies in Factors V, VIII, IX, X, XI, and XII. The breakdown of these associations included FVII deficiency with Factor V in three cases, Hemophilia A (factor VIII deficiency) in four cases, Hemophilia B (Factor IX deficiency) in seven cases, Factor X deficiency in five cases, and Factor XI deficiency in three cases.

In our study, focusing on a female cohort, we identified occurrences of all these combinations as well. However, given the significantly smaller sample size in the review of Girolami et al., drawing a prevalence comparison between their findings and our cohort is not feasible or relevant. This underlines the uniqueness of our study in providing insights into these rare combinations of bleeding disorders in a larger population.

In their 2007 study, Girolami et al. [18] reported associations between factor VII (FVII) deficiency and various non-coagulation-related disorders. These included Dubin–Johnson syndrome, Gilbert syndrome, cleft palate, deafness, hypospadia, intellectual disability, microcephaly, and carotid body tumors. However, in our cohort, none of these conditions were observed. Interestingly, the study by Girolami et al. did not report any cases associated with Noonan Syndrome either.

Given the rarity of FVII deficiency—though it is considered the least rare among the rare inherited bleeding disorders [19]—there is a lack of prevalence data on its combinations with other inherited bleeding disorders. The information we have about these associations mainly comes from cohort studies and literature reviews. This gap in knowledge underscores the need for more extensive research in this area to better understand the implications of FVII deficiency in the presence of other inherited disorders.

In 2021, a significant cohort study by Ahmadi et al. [20] included 450 patients with congenital combined bleeding disorders (CBDs), all referred to the Iranian Comprehensive Hemophilia Care Center (ICHCC) between 2010 and 2020. Within this group, the most prevalent CBDs identified were combined FV deficiency with FVII deficiency and FVII deficiency combined with FX deficiency, each accounting for 6 cases and representing 18.1% of the patients with CBDs in their cohort [20].

This finding contrasts with our cohort, where FVII deficiency was most frequently associated with VWD, accounting for 11.34% of all patients. Additionally, FV Leiden emerged as the second most common coagulation disorder associated with FVII deficiency in our cohort, comprising 4.22% of the cases.

Girolami et al. (2007) suggested differentiating between two types of associations of different bleeding disorders: Type I are independently evolving chance associations with other inherited bleeding disorders, and in Type II, there seems to be a common gene defect. Girolami et al. (2007) found examples for both types in Hemophilia A and B in their literature review [18].

This study has several limitations, including the following: (i) Selection Bias: there is a potential bias due to factors such as age at onset and gender. (ii) Patient Population: The study’s cohort consisted solely of white subjects. Consequently, the results may not be applicable to other racial groups, different age cohorts, or to patients with F7-associated bleeding who suffer from other fundamental diseases not covered in our cohort, like malignancies. (iii) Patient Ascertainment and Referral Bias: The patients in this study were continuously admitted from 2010 onwards and included both inpatients and outpatients. This period saw an increased awareness of bleeding symptoms in women and advancements in diagnostic and imaging techniques. Although the results at the initial onset of bleeding are independent of these changes in medical practices and the distribution of F7 mutations aligns with previous findings, there is still a possibility that the inclusion of both in- and outpatients might have introduced a referral bias, although this seems unlikely.

In conclusion, our cohort data reveal that the clinical phenotype in women with factor VII deficiency is predominantly influenced by factors such as age, blood group (BG), and the presence of other bleeding defects rather than solely by conventional genotyping methods. Consequently, for a more comprehensive scientific understanding, it is recommended to conduct further large-scale cooperative studies. These studies should employ advanced genetic approaches such as Genome-Wide Association Studies (GWAS) or exome studies to conduct in-depth investigations into the genetic underpinnings of this condition.

## Figures and Tables

**Table 1:** Novel genetic sequence variations (n = 30) found in this study (not listed in the EAHAD factor VII variant database or the scientific literature).

Sequence Change	Location	Amino Acid Change	Mutation	N of Alleles
c.-248C>T	Promoter		snv	1
c.-5_-4delinsCA	5′UTR		splice site	1
c.58G>A	Exon 1		missense	1
c.64+2T>C	Intron 1		splice site	1
c.98G>C	Exon 2	p.(Arg33Pro)	missense	1
c.156C>G	Exon 3	p.(His52Gln)	missense	1
c.170G>A	Exon 3	p.(Arg57Gln)	missense	1
c.218T>C	Exon 3	p.(Leu73Pro)	missense	1
c.256_257delinsTT	Exon 3	p.(Glu86Leu)	missense	2
c.265G>C	Exon 3	p.(Glu89Gln)	missense	1
c.291+2T>G	Intron 3		snv	1
c.291+5G>T	Intron 3		snv	1
c.291+71A>G	Intron 3		snv	1
c.292-26C>G	Intron 3		snv	2
c.431-40C>T	Intron 5		snv	1
c.431-7T>G	Intron 5		splice site	1
c.430C>G	Exon 5	p.(His144Asp)	missense	1
c.430C>T	Exon 5	p.(His144Tyr)	missense	1
c.475G>A	Exon 6	p.(Glu159Lys)	missense	1
c.632G>A	Exon 7	p.(Gly211Asp)	missense	1
c.646G>C	Exon 7	p.(Gly216Arg)	missense	1
c.664G>C	Exon 7	p.(Gly222Arg)	missense	2
c.682-9C>T	Intron 7		splice site	1
c.691_693del	Exon 8	p.(Leu231del)	deletion	1
c.718G>T	Exon 8	p.(Gly240Trp)	missense	1
c.797C>T	Exon 8	p.(Ala266Val)	missense	1
c.806-10T>C	Intron 8		snv	1
c.887C>T	Exon 9	p.(Pro296Leu)	missense	1
c.1204G>T	Exon 9	p.(Gly402Trp)	missense	2
c.1245G>A	Exon 9	p.(Thr415Thr)	synonymous	1

snv: single-nucleotide variant (non-coding; non-splice site).

**Table 2:** (**a**) Numbers and percentages of patients with VWD as secondary and/or further bleeding disorders. (**b**) Numbers and percentages of patients with disorders other than VWD as secondary and/or further bleeding disorders.

(**a**)					
**Additional Bleeding Disorders**			**Number of Patients**	**% of Patients**	
**Second Bleeding Disorder**	**Third Bleeding Disorder/Factor Deficiency**	**Fourth Bleeding Disorder/Factor Deficiency**		**785 with F VII Deficiency**	**193 with Additional Bleeding Disorders**
VWD only			70	9.92	36.27
VWD plus	Noonan syndrome		1	0.13	0.52
	Hyperhomocyteinemia				
	F I		4	0.51	2.07
	F V		1	0.13	0.52
	F VIII				
	F IX		2	0.25	1.04
	F X				
	F XII				
	F XIII		4	0.51	2.07
	F I plus	F V	1	0.13	0.52
VWD total			89	11.34	46.11
(**b**)					
**Additional Bleeding Disorders**			**Number of Patients**	**% of Patients**	
**Second Bleeding Disorder**	**Third Bleeding Disorder/Factor Deficiency**	**Fourth Bleeding Disorder/Factor Deficiency**		**785 with F VII Deficiency**	**193 with Additional Bleeding Disorders**
F VIII only			2	0.25	1.04
F VIII plus	F XIII		1	0.13	0.52
F VIII plus	VWS				
F VIII total			4	0.51	2.07
F IX only			1	0.13	0.52
F IX plus	Noonan syndrome				
F IX plus	VWS		2	0.25	1.04
F IX total			3	0.38	1.55
F I only			8	1.02	4.13
F I plus	F X	F XII	1	0.13	0.52
F I total			9	1.15	4.66
F II only/total			1	0.13	0.52
F V only			11	1.40	5.70
F V plus	F 1		1	0.13	0.52
F V total			12	1.53	6.22
F X only			17	2.17	8.81
F X plus	F XII		1	0.13	0.52
F X total			18	2.29	9.33
F XI only/total			6	0.76	3.11
F XII only/total			26	3.31	13.47
F XIII only/total			13	1.66	6.74
Fibrinogen only/total			11	1.40	5.70
Plasminogen only/total			1	0.13	0.52
Additional further bleeding disorders other than VWD as secondary bleeding disorder total			104	13.25	53.89

F: factor; VWD: von Willebrand disease.

## Supplementary Materials

The following supporting information can be downloaded at: https://www.mdpi.com/article/10.3390/jcm13010049/s1, Table S1. Total genetic sequence variations (n = 354) found in this study (bold variants were not to be found in EAHAD F7-database or scientific literature; snv = single nucleotide variant [non-coding, non splice site]). Supplementary Table S2. Numbers and percentages of patients with additional thrombophilia.
