# Supplementary material for: Genotype–Phenotype Relationship among 785 Unrelated White Women with Inherited Congenital Factor VII Deficiency: A Three-Center Database Study
Source: J Clin Med. 2023 Dec 21;13(1):49. doi: 10.3390/jcm13010049 (PMC10779797; doi:10.3390/jcm13010049)
Supplement: Supplementary file 1 [file jcm-13-00049-s001.zip › jcm-2671474-supplementary.pdf]

Journal of Clinical Medicine

MDPI

Suplementary tables

Supplementary Table S1. Total genetic sequence variations (n=354) found in this study (bold variants were not to be found in EAHAD F7-database or scientific literature; snv = single nucleotide variant [non-coding, non splice site])

Sequence change	Location	Amino acid change	Mutation	N of alleles
**c.-248C>T**	**Promoter**		**snv**	**1**
c.-89C>T	Promoter		snv	1
c.-65G>C	Promoter		snv	2
c.-55C>G	Promoter		snv	2
c.-55C>T	Promoter		snv	5
c.-44T>C	1 (5'UTR)		snv	6
c.-30A>C	1 (5'UTR)		snv	4
**c.-5_-4delinsCA**	**1 (5'UTR)**		**splice site**	**1**
c.14C>A	1	p.(Ala5Asp)	missense	2
c.56T>C	1	p.(Leu19Pro)	missense	1
**c.58G>A**	**1**	**p.(Ala20Thr)**	**missense**	**1**
c.64G>A	1	p.(Gly22Ser)	missense	4
**c.64+2T>C**	**1 (Intron)**		**splice site**	**1**
**c.98G>C**	**2**	**p.(Arg33Pro)**	**missense**	**1**
**c.156C>G**	**3**	**p.(His52Gln)**	**missense**	**1**
**c.170G>A**	**3**	**p.(Arg57Gln)**	**missense**	**1**
c.178C>G	3	p.(Arg60Gly)	missense	1
c.208C>T	3	p.(Pro70Ser)	missense	1
c.215C>G	3	p.(Ser72Cys)	missense	2
c.218T>A	3	p.(Leu73Gln)	missense	2
**c.218T>C**	**3**	**p.(Leu73Pro)**	**missense**	**1**
c.220dupG	3	p.(Glu74Glyfs*33)	frameshift	3
c.235G>C	3	p.(Glu79Gln)	missense	2
c.245G>T	3	p.(Cys82Phe)	missense	2
c.247T>C	3	p.(Ser83Pro)	missense	1
**c.256_257delinsTT**	**3**	**p.Glu86Leu**	**missense**	**2**
c.262C>G	3	p.(Arg88Gly)	missense	2
**c.265G>C**	**3**	**p.(Glu89Gln)**	**missense**	**1**
c.291+1G>C	3 (Intron)		snv	3
**c.291+2T>G**	**3 (Intron)**		**snv**	**1**
**c.291+5G>T**	**3 (Intron)**		**snv**	**1**
**c.291+71A>G**	**3 (Intron)**		**snv**	**1**
**c.292-26C>G**	**3 (Intron)**		**snv**	**2**
c.317-1G>C	4 (Intron)		splice site	2

c.430+1G>A	5 (Intron)		splice site	2
**c.431-40C>T**	**5 (Intron)**		**snv**	**1**
**c.431-7T>G**	**5 (Intron)**		**splice site**	**1**
**c.430C>G**	**5**	**p.(His144Asp)**	**missense**	**1**
**c.430C>T**	**5**	**p.(His144Tyr)**	**missense**	**1**
c.460G>A	6	p.(Glu154Lys)	missense	3
c.466G>A	6	p.(Gly156Ser)	missense	4
c.469G>A	6	p.(Gly157Ser)	missense	2
**c.475G>A**	**6**	**p.(Glu159Lys)**	**missense**	**1**
c.479A>G	6	p.(Gln160Arg)	missense	5
c.571+78G>A	6 (Intron)		snv	2
c.580C>A	7	p.(Pro194Thr)	missense	1
c.583T>C	7	p.(Cys195Arg)	missense	2
c.589A>G	7	p.(Lys197Glu)	missense	4
**c.632G>A**	**7**	**p.(Gly211Asp)**	**missense**	**1**
c.634C>T	7	p.(Arg212*)	nonsense	2
c.635G>A	7	p.(Arg212Gln)	missense	2
**c.646G>C**	**7**	**p.(Gly216Arg)**	**missense**	**1**
**c.664G>C**	**7**	**p.(Gly222Arg)**	**missense**	**2**
**c.682-9C>T**	**7 (Intron)**		**splice site**	**1**
**c.691_693del**	**8**	**p.(Leu231del)**	**deletion**	**1**
**c.718G>T**	**8**	**p.(Gly240Trp)**	**missense**	**1**
c.791T>C	8	p.(Leu264Pro)	missense	1
c.796G>A	8	p.(Ala266Thr)	missense	1
**c.797C>T**	**8**	**p.(Ala266Val)**	**missense**	**1**
c.805+3_805+6del	8 (Intron)		splice site	2
c.805+7A>G	8 (Intron)		splice site	29
**c.806-10T>C**	**8 (Intron)**		**snv**	**1**
c.814G>A	9	p.(Asp272Asn)	missense	2
c.817_831del	9	p.(Leu273_Asp277del)	deletion	10
c.829G>A	9	p.(Asp277Asn)	missense	1
c.847C>T	9	p.(Arg283Trp)	missense	2
c.851G>A	9	p.(Arg284Gln)	missense	2
**c.887C>T**	**9**	**p.(Pro296Leu)**	**missense**	**1**
c.910G>A	9	p.(Ala304Thr)	missense	1
c.911C>T	9	p.(Ala304Val)	missense	13
c.920G>A	9	p.(Arg307His)	missense	1
c.934G>A	9	p.(Val312Met)	missense	9
c.968T>C	9	p.(Leu323Pro)	missense	2
c.973G>A	9	p.(Glu325Lys)	missense	3
c.985T>C	9	p.(Ser329Pro)	missense	1

c.1009C>T	9	p.(Arg337Cys)	missense	2
c.1027G>A	9	p.(Gly343Ser)	missense	2
c.1061C>T	9	p.(Ala354Val)	missense	87
c.1074G>A	9	p.(Met358Ile)	missense	2
c.1088C>G	9	p.(Pro363Arg)	missense	18
c.1090C>T	9	p.(Arg364Trp)	missense	2
c.1091G>A	9	p.(Arg364Gln)	missense	4
c.1151C>T	9	p.(Thr384Met)	missense	4
c.1160T>C	9	p.(Met387Thr)	missense	2
c.1163T>C	9	p.(Phe388Ser)	missense	1
c.1171G>A	9	p.(Gly391Ser)	missense	9
**c.1204G>T**	**9**	**p.(Gly402Trp)**	**missense**	**2**
c.1224T>G	9	p.(His408Gln)	missense	1
**c.1245G>A**	**9**	**p.(Thr415=)**	**synonymous**	**1**
c.1256C>T	9	p.(Thr419Met)	missense	1
c.1310A>T	9	p.(Tyr437Phe)	missense	3
c.1384C>T	9	p.(Arg462*)	nonsense	2
c.1391delC	9	p.(Pro464Hisfs*32)	frameshift	21
c.1_1401del	1 - 9	p.(Met1?)	complete deletion	2

Supplementary Table S2. Numbers and percentages of patients with additional thrombophilia

**Thrombophilia type**				**Number of patients**	**% of patients**	
**second**	**third**	**fourth**	**fifth**		all 785	all 189 with additional thrombophilia
F V Leiden only				34	4,33	17,99
F V Leiden plus	Prothrombin G20210A mutation			1	0,13	0,53
	Prothrombin A19911G mutation			2	0,25	1,06
	Prothrombin A19911G mutation plus	MTHFR C677T Mutation				
	Prothrombin A19911G mutation plus	MTHFR C677T mutation plus	Lipoprotein a metabolism disorder	1	0,13	0,53
	MTHFR C677T mutation plus		PAI 4G/4G mutation			
**F V Leiden total**				**41**	**5,22**	**21,69**
Prothrombin G20210A mutation only				27	3,44	14,29
Prothrombin G20210A mutation plus		Enhanced F VIII		2	0,25	1,06
		Lipoprotein a metabolism disorder		1	0,13	0,53
		MTHFR C677T mutation plus	PAI 4G/4G mutation			
**Prothrombin G20210A mutation total**				**32**	**4,08**	**16,93**
Prothrombin A19911G only				17	2,17	8,99
Prothrombin A19911G plus		Lipoprotein a metabolism disorder		1	0,13	0,53
		Enhanced F VIII				
		MTHFR C677T mutation				
		MTHFR C677T mutation plus	PAI 4G/4G mutation			
**Prothrombin A19911G total**				**21**	**2,68**	**11,11**
MTHFR C677T only				20	2,55	10,58
MTHFR C677T plus		Lipoprotein a metabolism disorder		3	0,38	1,59
		Protein S-, C- or antithrombin deficiency		1	0,13	0,53
**MTHFR C677T total**				**24**	**3,06**	**12,70**

**Thrombophilia type**				**Number of patients**	**% of patients**	
**second**	**third**	**fourth**	**fifth**		all 785	all 189 with additional thrombophilia
PAI 4G/4G				6	0,76	3,17
Lipoprotein a metabolism disorder				28	3,57	14,81
Protein S-, C- or antithrombin deficiency				19	2,42	10,05
Antiphospholipid syndrome				2	0,25	1,06
Enhanced FVIII				3	0,38	1,59
